# Ki67 expression levels are a better marker of reduced melanoma growth following MEK inhibitor treatment than phospho-ERK levels

**DOI:** 10.1038/sj.bjc.6603596

**Published:** 2007-01-23

**Authors:** K S M Smalley, R Contractor, N K Haass, J T Lee, K L Nathanson, C A Medina, K T Flaherty, M Herlyn

**Affiliations:** 1 The Wistar Institute, 3601 Spruce Street, Philadelphia, Pennsylvania, 19104, USA; 2 Division of Medical Genetics, University of Pennsylvania School of Medicine, Philadelphia, PA, USA; 3 Hematology-Oncology, University of Pennsylvania School of Medicine, Philadelphia, PA, USA; 4 Abramson Cancer Center of the University of Pennsylvania, Philadelphia, PA 19104, USA

**Keywords:** melanoma, Ki67, biomarker, BRAF, MAP kinase, Akt

## Abstract

The loss of tumour phospho-extracellular responsive kinase (pERK) positivity is the major treatment biomarker for mitogen-activated protein kinase/extracellular responsive kinase (MEK) inhibitors. Here, we demonstrate that there is a poor correlation between pERK inhibition and the anti-proliferative effects of MEK inhibitors in melanoma cells. We suggest that Ki67 is a better biomarker for future clinical studies.

Constitutive activity of BRAF is known to be critical for the proliferation and survival of melanoma cells through the activation of the RAF/MEK/ERK mitogen activated protein (MAP) kinase pathway (Smalley, 2003). In light of this, a number of research groups and pharmaceutical companies have begun to explore whether targeting the BRAF/MEK pathway using shRNA and small molecule inhibitors are viable therapeutic approaches (Hingorani *et al*, 2003; Karasarides *et al*, 2004; Sharma *et al*, 2005). In a recent series of clinical trials of MEK inhibitors, investigators have used tumour phospho-ERK (pERK) levels as a biomarker (Rinehart *et al*, 2004; Lorusso *et al*, 2005). These studies have demonstrated that although the MEK inhibitors, such as CI-1040 and PD0325901, inhibit constitutive pERK activity within tumours there was little clinical activity. The obvious conclusion is that these agents, like so many others, are ineffective in the clinical setting. However, there is an alternate explanation—the concentrations of MEK inhibitor required to inhibit the pathway are not correlated with those required to inhibit cell growth. The link between pERK levels and inhibition of growth has not been explored in melanoma. In the current study, we have studied two MEK inhibitors and investigated the relationship between the concentrations required to inhibit pERK and those required to block the growth of a panel of melanoma cell lines.

## MATERIALS AND METHODS

### Cell culture

Human melanoma cells were isolated and cultured as described in Smalley *et al* (2005).

### Adherent cell proliferation analysis

Cells were treated with increasing concentrations of U0126 (Calbiochem, San Diego, CA, USA) (0.01–30 *μ*M) CI-1040 or LY294002 in triplicate. Methylthiazolyldiphenyl-tetrazolium bromide (MTT) assays were performed as described previously (Smalley *et al*, 2006).

### Western-blot analysis

Proteins were extracted and blotted as described in Smalley *et al* (2005). Antibodies to pERK, total ERK (tERK), phospho-Akt and Akt were from Cell Signaling Technology (Beverly, MA, USA).

### Ki67 staining and quantification

Melanoma cells were prepared and stained as described previously (Smalley *et al*, 2005). The primary antibody (anti-Ki67) was from Zymed (San Franscisco, CA, USA). Cells were counterstained with DAPI. Cells from six high-powered fields (× 60) were counted and the number of Ki67-positive cells were expressed as a percentage of total cell number (DAPI-positive).

### CGH and BRAF/N-Ras sequencing

Comparative genomic hybridization (CGH) and mutation sequencing were performed as described previously (Hoek *et al*, 2006). The mutational status of the C8161 was reported in Sharma *et al* (2005).

### Cell cycle analysis

Cell cycle analysis was performed after treatment with U0126 or CI-1040 as described in Smalley *et al* (2005).

### Analysis

Unless otherwise stated, all data show the mean of at least three independent experiments. Where appropriate, the data show the mean±s.e.

## RESULTS

### Most melanoma cell lines in our panel are BRAF V600E mutant, N-Ras wt, c-Kit wt

The panel of seven melanoma cell lines were sequenced for BRAF, N-Ras and c-Kit mutations (Table 1). The panel of cell lines chosen represented the heterogeneity of mutations found in human melanomas. Thus, most of the cell lines tested (5/7 or 70%) harboured the V600E mutation in BRAF and were heterozygous for the BRAF mutation. The two exceptions were C8161, which was wild-type for both BRAF and N-Ras mutations (1/7 or 15%), and SbCl2, which harbours the Codon-61 N-Ras mutation (1/7 or 15%). None of the cell lines tested had mutations in c-Kit.

### Lack of strict correlation between pERK inhibition and reduced cell proliferation on a panel of melanoma cell lines

**Figure 1:**
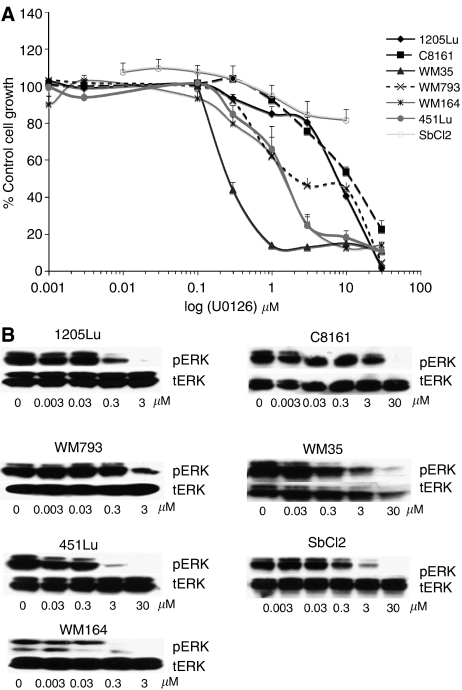
**U0126 inhibits pERK and growth of a panel of melanoma cells** (**A**) Cells were treated with increasing concentrations of U0126 (1 nM–30 *μ*M) for 72 h before being treated with MTT. Absorbances were read at 570 nm and expressed as a percentage of control absorbance. Data show the mean of three independent experiments ±s.e.m. (**B**) Reduction of pERK activity following U0126 treatment. Cells were treated with U0126 for 24 h and probed for pERK. Blots were stripped and reprobed for tERK to demonstrate equal protein loading.

Treatment of melanoma cells with increasing concentrations of U0126 led to inhibition of growth in all cell lines apart from the SbCl2. There was a great deal of variation between the IC_50_ values of U0126 on the melanoma cell lines that ranged from 300 nM (for WM35) to 10 *μ*M (for C8161) (Figure 1A). When we compared the concentrations of U0126 needed to inhibit pERK levels with those required to inhibit growth we saw little correlation. WM35 cells, which had the lowest IC_50_ for growth inhibition (280 nM), did not show substantial reduction in pERK phosphorylation until 3 *μ*M U0126 (Figure 1B). In the SbCl2 cell line, pERK was inhibited in the absence of any effects upon cell growth. Whereas in the 1205Lu cells, pERK was inhibited at 300 nM U0126 and there was little cell growth inhibition until 10 *μ*M U0126 (Figure 1B). To explore whether these findings were the result of poor compound stability in tissue culture, we treated the 1205Lu cells with U0126 for 48 and 72 h and found that pERK levels were still blocked (data not shown).

### Increasing concentrations of U0126 increases G1 phase cell cycle block and reduces Ki67 staining

**Figure 2:**
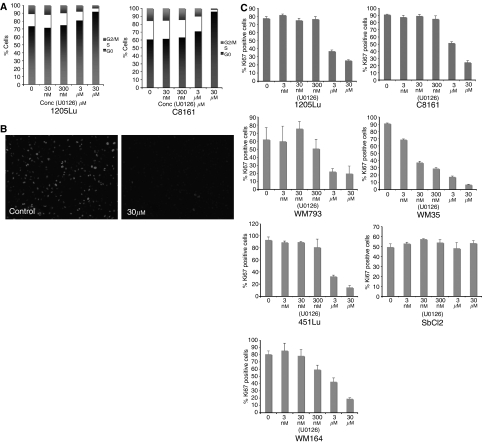
**U0126 reduces S-phase fraction of melanoma cells and reduces Ki67 staining.** (**A**) Cells treated with U0126 (30 nM–30 *μ*M) for 24 h were found to undergo G1-phase cell cycle arrest. (**B**) Representative picture showing reduction in Ki67 staining of 1205Lu cells following U0126 (30 *μ*M) treatment. (**C**) Quantification of Ki67 staining following U0126 treatment. Cells were treated with U0126 for 24 h and stained for both Ki67 and DAPI. Data show mean of three experiments where six high-power fields were counted. Ki67-positive cells were expressed as a percentage of total cell number (DAPI staining).

Having demonstrated that pERK levels did not correlate with inhibition of melanoma cell growth we next explored whether there was a better correlation with expression of the cell proliferation marker Ki67. Cell cycle analysis demonstrated that increasing concentrations of U0126 reduced the proportion of cells undergoing S-phase transition (Figure 2A). In these instances, there was a closer link between reduction in S-phase fraction and inhibition of growth (Figure 1A). Treatment of the melanoma cells with U0126 reduced the percentage of cells staining positively for Ki67 (Figure 2B,C). Quantification of these results revealed a parallel between the concentrations of U0126 required to inhibit cell growth and those required to reduce the fraction of Ki67-positive cells (Figure 2C). In particular, a striking reduction of Ki67 staining was noted for the WM35 cell line when treated with 300 nM U0126 (Figure 2C), even though high pERK levels were maintained (Figure 1B). In another prominent example, the SbCl2 cell line was found to be poorly responsive to the growth inhibitory effects of U0126 (Figure 1A), even though pERK levels were inhibited (Figure 1B). The lack of growth inhibition was reflected in the levels of Ki67 staining, which were not diminished at the highest concentration of U0126 (Figure 2C).

### The MEK inhibitor CI-1040 has similar effects to that of U0126 on pERK levels and cell growth

**Figure 3:**
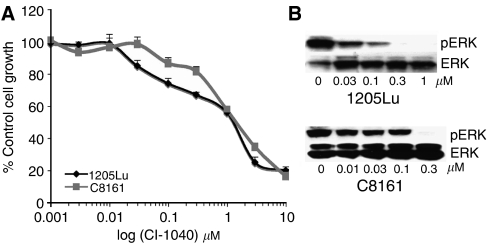
**CI-1040 inhibits pERK and growth of two melanoma cell lines** (**A**) Cells were treated with increasing concentrations of CI-1040 (1 nM–10 *μ*M) for 72 h before being treated with MTT. Absorbances were read at 570 nm and expressed as a percentage of control absorbance. Data show the mean of three independent experiments ±s.e.m. (**B**) Reduction of pERK activity following CI-1040 treatment. Cells were treated with U0126 for 24 h and probed for pERK. Blots were stripped and reprobed for tERK to demonstrate equal protein loading.

Although U0126 is a useful tool compound it is not clinically relevant. To confirm that similar responses were seen with a clinically relevant MEK inhibitor, we treated the C8161 and 1205Lu cells with increasing concentrations of CI-1040. It was noted that CI-1040 was more potent than U0126 at reducing the growth of the C8161 and 1205Lu cell lines (Figure 3A). Again, like U0126, there was a disconnection between the concentrations required to inhibit growth and those to inhibit pERK levels (Figure 3B). For the 1205Lu cells, pERK levels were substantially reduced at 30 nM, with complete loss of all pERK activity at 300 nM. In the MTT assay, there was only 33% reduction in 1205Lu cell growth at 300 nM CI-1040 (Figure 3A,B). Likewise, for the C8161 cells, all pERK activity was blocked at 300 nM CI-1040, which only corresponded to an 18% inhibition of cell growth (Figure 3A,B).

### Inhibition of growth following PI3 kinase inhibitor treatment correlates better with Ki67 staining than inhibition of pAKT levels

**Figure 4:**
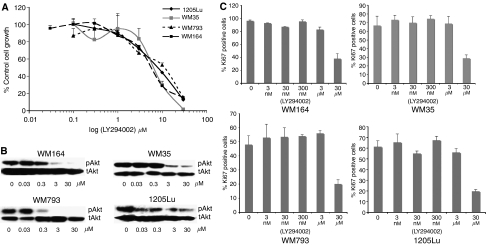
**LY294002-mediated inhibition of melanoma cell growth also correlates with reduced Ki67 staining** (**A**) Cells were treated with increasing concentrations of LY294002 (30 nM–30 *μ*M) for 72 h before being treated with MTT. Absorbances were read at 570 nm and expressed as a percentage of control absorbance. Data show the mean of three independent experiments ±s.e.m. (**B**) Reduction of phospho-Akt activity following LY249002 treatment. Cells were treated with LY294002 for 24 h and probed for pAkt. Blots were stripped and reprobed for total Akt to demonstrate equal protein loading. (**C**) Quantification of Ki67 staining following LY294002 treatment. Cells were treated with LY294002 for 24 h and stained for both Ki67 and DAPI. Data show mean of three experiments where six high-power fields were counted. Ki67-positive cells were expressed as a percentage of total cell number (DAPI staining).

To determine whether the results seen with MEK inhibition were applicable to other signalling pathways active in melanoma, we next focused upon the PI3K/Akt pathway. Treatment of melanoma cells with increasing concentrations of the PI3 kinase inhibitor LY294002 led to a concentration-dependent reduction in cell growth (Figure 4A). Unlike U0126, there was little difference in the IC_50_ for LY294002 between the cell lines, which was around 10 *μ*M. Analysis of LY294002-mediated inhibition of pAkt levels showed some variation between the cell lines. Nearly all of the pAkt was inhibited at 300 nM in the WM793 cell lines, whereas substantial levels of pAkt were observed in the 1205Lu cells even after treatment with 30 *μ*M LY294002 (Figure 4B). In agreement with our results on U0126, there was a good correlation between LY294002-mediated inhibition of cell growth (Figure 4A) and reduction in Ki67 staining (Figure 4C).

## DISCUSSION

Inhibitors which target the BRAF/MAP kinase pathway are currently under intense preclinical and clinical investigation in melanoma. One of the most common biomarkers used is the level of pERK tumour biopsies. A number of recent studies have shown that although pERK levels are indeed blocked in the lesions of patients undergoing CI-1040 treatment, there is little clinical benefit (Rinehart *et al*, 2004; Lorusso *et al*, 2005). This raises the question of whether inhibition of pERK is correlated with the anti-proliferative activities of the MEK inhibitors in this setting.

Here, we demonstrate that there is little parallel between the inhibition of pERK and the anti-proliferative activity of two MEK inhibitors in a panel of seven melanoma cell lines. Some cell lines, such as WM35 and C8161, demonstrate reduced cell growth in the relative absence of pERK inhibition, whereas others such as 1205Lu and SbCl2 demonstrate reduced pERK levels in the absence of growth inhibition. There was no link between these discrepancies and BRAF mutational status as nearly all (five out of seven) of these melanoma lines harboured the V600E mutation in BRAF. Others have demonstrated the presence of amplifications in downstream components of the cell cycle machinery such as cyclin D1 and CDK4, which may contribute to this observed resistance (Curtin *et al*, 2005; Muthusamy *et al*, 2006). However, CGH analysis of our panel of seven melanoma cell lines did not reveal amplifications in cyclin D1 or CDK4 (data not shown). A more probable explanation for these results is the high constitutive activity in other signalling pathways that also drive melanoma cells through the cell cycle, such as PI3K/Akt and Src/STAT3. It is possible that there is some functional redundancy between the mitogenic pathways in melanoma cells, so that inhibition of one pathway, such as MAP kinase, may not adversely impact on melanoma cell growth.

The effects of MEK inhibitors upon melanoma cells are mainly cytostatic and we see little evidence of apoptosis induction. In line with this observation, there is a better correlation with growth of the U0126-induced anti-proliferative effects and the reduction of Ki67 staining. As a similar correlation is seen using other putative targeted therapy agents, such as PI3 kinase inhibitors, it is therefore suggested that Ki67 staining of tumour sections could be a general strategy for following the progress of targeted therapy trials in melanoma. The implications of this study are clear; although the use of phospho-specific antibody staining as a biomarker may give some measure of target inhibition, it may not necessarily correlate with the desired end biological effect (e.g. growth inhibition or apoptosis). The planning of future clinical trials would likely benefit from the inclusion of both a phospho-specific signal transduction marker (such as pERK) and a proliferation marker (such as Ki67) to allow a true measure of treatment progress at the level of the tumour.

## Figures and Tables

**Table 1:** Mutational profiles of BRAF, N-Ras and c-Kit in the panel of melanoma cells

**Cell line**	**Lesion type**	**BRAF**	**N-Ras**	**c-Kit**	**BRAF digest**
WM35	RGP	V600E	Wt	wt	HET
SBCl2	RGP	Wt	61K	wt	Wt
WM793	VGP	V600E	Wt	wt	HET
1205Lu	Met	V600E	Wt	wt	HET
451Lu	Met	V600E	Wt	wt	HET
WM164	Met	V600E	Wt	wt	HET
C8161	Met	Wt	Wt	ND	ND

Wt, wild-type, ND, not determined, HET, heterozygous mutation.
